# Learning Curve for D2 Lymphadenectomy in Gastric Cancer

**DOI:** 10.1155/2013/508719

**Published:** 2013-06-16

**Authors:** Alexis Luna, Pere Rebasa, Sandra Montmany, Salvador Navarro

**Affiliations:** Department of General Surgery, Hospital Universitari Parc Taulí, Sabadell, Catalonia, Spain

## Abstract

*Background*. D2 lymphadenectomy is a demanding technique which is associated with high morbidity in the West. We report our experience with D2 lymphadenectomy after a training period in Japan. *Methods*. Prospective, descriptive study in 133 consecutive patients undergoing radical gastrectomy for gastric adenocarcinoma from 2005 to 2011. We analysed the number of lymph nodes removed, observed morbidity/mortality compared with the predictions of POSSUM and O-POSSUM, survival, and disease-free interval for patients with D1 and D2 lymphadenectomy. *Results*. The morbidity rate in patients with D1 lymphadenectomy was 59.4%. For D2 it was 47.7%. The mortality rate in patients with D1 was 6.7%. In the D2 group it was 6.8%. Median survival was 42.9 months in D1 and 55 months in D2. The disease-free interval was 49 months for D1 and 58 months for D2. *Conclusion*. The learning curve for D2 lymphadenectomy presents acceptable rates of morbidity and mortality, providing that the technique is learnt at a center with extensive experience.

## 1. Introduction

Deciding on the type of lymphadenectomy to perform in gastric cancer is a controversial matter. Groups in Asia contend that lymph node involvement in gastric cancer in the absence of distant metastases is a localized disease and that curative surgical treatment should be performed. They advocate D2 lymphadenectomy on the grounds that it improves staging and locoregional control of disease. In the West, lymph node involvement tends to be considered a systemic disease with low likelihood of cure. In the last two decades, the literature has shown that the results of gastric cancer surgery are far better in Asia, where D2 lymphadenectomy is considered the standard five-year survival rates in Japan are around 50–60% [1], compared with the figures of 10–30% reported in the West [2].

D2 lymphadenectomy is a demanding technique, especially in patients with higher body mass index, as is generally the case in the West. Patients in our setting also tend to be older and more likely to present comorbidity. As a result, D1 lymphadenectomy is considered the standard in the West, although certain groups now advocate D2 lymphadenectomy [3]. The evidence available to compare D1 and D2 lymphadenectomy is limited and has serious shortcomings. Most of the literature consists of retrospective cohort studies with substantial bias; only two methodologically sound, prospective, randomized, and multicenter studies have been published, but both present serious problems regarding treatment selection. Studies performed in Japan are retrospective and observational [2], and no prospective, randomized studies have been carried out in that country to date. So far, no overall increase in survival with D2 lymphadenectomy has been demonstrated in comparison with D1, although patients at stages IIa and III appear to benefit from D2 [4]. Because of their low operative mortality (below 2%) and the good survival results, Japanese surgeons see no need for prospective randomized studies comparing D1 and D2.

The well-designed studies by Hartgritik et al. and Cuschieri et al. [5, 6] provide the strongest evidence regarding the utility of D2 lymphadenectomy but present major biases such as lack of prior training to testing. Although the learning curve for D2 lymphadenectomy is estimated to be 25 procedures, many of the surgeons in these two studies did not meet this criterion. The need to perform splenic and pancreatic resections, which are not routine procedures, also skews the morbidity and mortality presented in these studies.

The technique of D2 lymphadenectomy has evolved considerably in recent years. Neither distal pancreatectomy nor splenectomy is currently included, due to the increase in morbidity and mortality and the lack of any clear prognostic benefit [7]. In D1+ lymphadenectomy the tail of the pancreas and spleen are preserved, and therefore groups 10 and distal 11 cannot be completely removed [8]. In our environment, the majority of surgeons consider this procedure to be a D2 lymphadenectomy. The resection of the tail of the pancreas and spleen is reserved for tumor invasion of these organs.

Members of our group spent a training period in Japan learning to perform gastrectomy with D2 lymphadenectomy, following reports of reduced morbidity and mortality by other groups from Italy [3] and Spain [9]. Reducing operative morbidity and mortality to the levels obtained with D1 lymphadenectomy is the key to adequate comparison of the survival of patients undergoing either technique.

## 2. Objective

To assess the evolution of the number of nodes removed, morbidity, mortality, and survival during the surgeons' learning curve for D2 lymphadenectomy in comparison with D1 lymphadenectomy performed in patients with gastric cancer.

## 3. Material and Methods

This is a prospective study that include 133 consecutive patients undergoing radical gastrectomy for resectable gastric adenocarcinoma from January 2005 to December 2011.

The extension of the gastrectomy was determined by the location of the tumor, with a margin of 5 cm. In D2 lymphadenectomy cases, resection of the spleen and pancreas was restricted to cases with local invasion or when lymph node involvement at levels 10 or 11d was suspected. Cytology of the peritoneal lavage was performed in all patients.

Node dissection in patients who underwent D2 lymphadenectomy was performed in accordance with the recommendations of the JRSGC [1].

Operative and postoperative complications and mortality were prospectively recorded at 30 days using an ACCESS database (Microsoft Corporation, Seattle, WA, USA) with a protected format [10]. Complications were graded according to the Clavien scale [11]. We were especially accurate and systematic with the registering of this data, as apart from collecting the variables, which are included as a precaution against risk; we collected all the adverse effects, which could have affected the patient (e.g., vein inflammation). All nosocomial, surgical, and nonsurgical complications were included.

Three members of the gastric surgery unit had spent training periods at the National Cancer Center in Tokyo (Japan) in October 2005 and March 2007 in order to broaden their knowledge of gastrectomy with D2 lymphadenectomy. Until 2005, all patients at our center had undergone D1 lymphadenectomy. After the training period, patients with BMI < 30, age < 70 years, without cardiac comorbidity (NYHA < II) or severe kidney disease (requiring dialysis) were selected for D2 lymphadenectomy. 

We analysed the number of lymph nodes removed, actual morbidity/mortality compared with the predictions of POSSUM [12] and O-POSSUM [13], survival, and disease-free interval.

The POSSUM [12] was measured postoperatively, immediately after entering the preoperative physiological variables and the surgical variables on a spreadsheet available at http://www.sfar.org/scores2/possum2.html. 

The O-POSSUM [13] was obtained by entering the preoperative physiological variables and the surgical variables on a spreadsheet available at http://riskprediction.org.uk/op-index.php.

## 4. Statistics

Categorical data are presented as percentages. Continuous data are presented as means and 95% confidence intervals. Given the study design, no attempt was made to compare between the two populations of patients undergoing D1 and D2; the data are described separately. However, Kaplan-Meier survival curves were plotted and compared by log rank, with the considerations noted in the Discussion. For the study of predictors of survival, also bearing in mind the restrictions imposed by the study design, we used Cox regression modeling the equation through the inclusion and sequential removal of variables which showed a *P* value <0.2 in the univariate analysis.

## 5. Results

During 2005 we performed 20 D1 gastrectomies, isolating a mean of 20.1 lymph nodes. After the training period, 69 D1 gastrectomies were performed; the mean number of nodes isolated rose to 23.7 (a 15.2% increase).

Since January 2006 we have performed 44 D2 gastrectomies, isolating a mean of 36.6 lymph nodes. In the first two years of the learning period (2006-2007) we performed 13 D2 gastrectomies, isolating a mean of 28.8 lymph nodes, whereas in 2008–2011 we performed 31 D2 gastrectomies with a mean of 39.8 lymph nodes. The number of lymph nodes removed rose by 27.6% over the period due to the increased experience of the surgical team and pathologists.

The morbidity rate among patients with D1 lymphadenectomies between January 2006 and December 2011 was 59.4% (POSSUM 49.4%, observed/expected morbidity ratio 1.202). For D2 lymphadenectomy the rate was 47.7% (POSSUM 33.7%, ratio 1.415) (Table 1). The observed and expected morbidity ratios showed 20% more complications in D2 than in D1. The complications are listed in Table 2, ranked by the impact they cause. There was a significant increase in serious surgical complications such as anastomotic leakage, intra-abdominal abscess, and evisceration in the D1 group. In the D2 group there was an increase in pancreas-related complications such as pancreatitis and pancreatic fistula. The only severe pancreatic fistula occurred in a patient who underwent pancreatectomy due to neoplastic infiltration. 

The rest of pancreatitis and pancreatic fistulas have been mild and easy to manage. We highlight the increase in number and severity of complications in the D1 group, probably attributable to the increased complexity and comorbidity of patients. 

The mortality rate in patients with D1 was 6.7% (6 patients, 13.9% O-POSSUM, and observed/expected morbidity ratio 0.482) and 6.8% in the D2 group (3 patients, 7.2% O-POSSUM, and ratio 0.944) (Table 1). The only death in the D2 group attributable to a surgical complication was due to pancreatitis after pancreatectomy for neoplastic infiltration in the patient mentioned above. 

**Figure 1:**
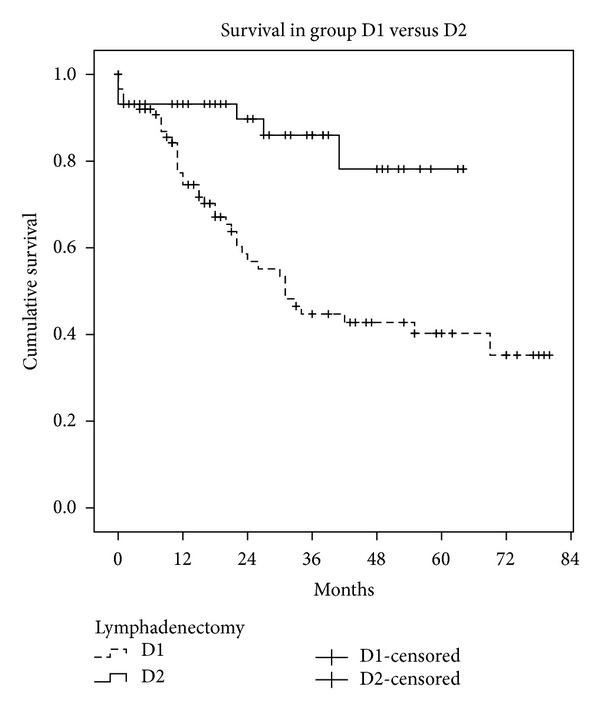
Survival in group D1 versus group D2.

**Figure 2:**
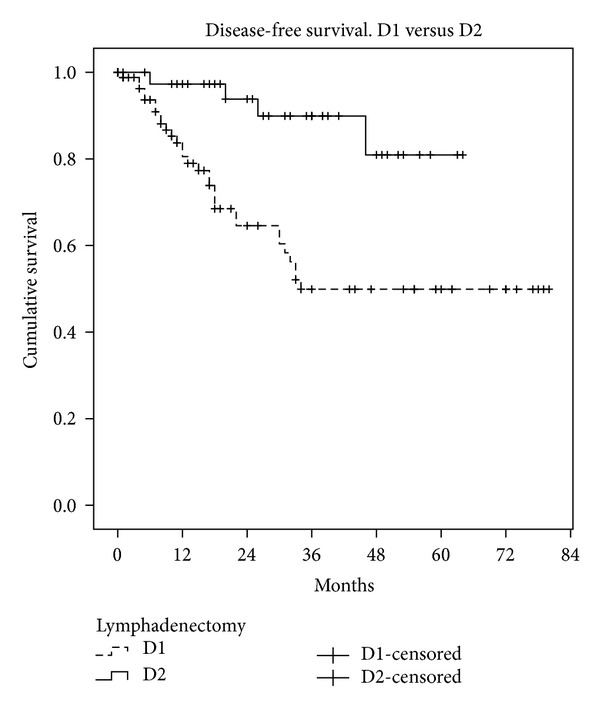
Disease-free interval, group D1 versus group D2.

**Figure 3:**
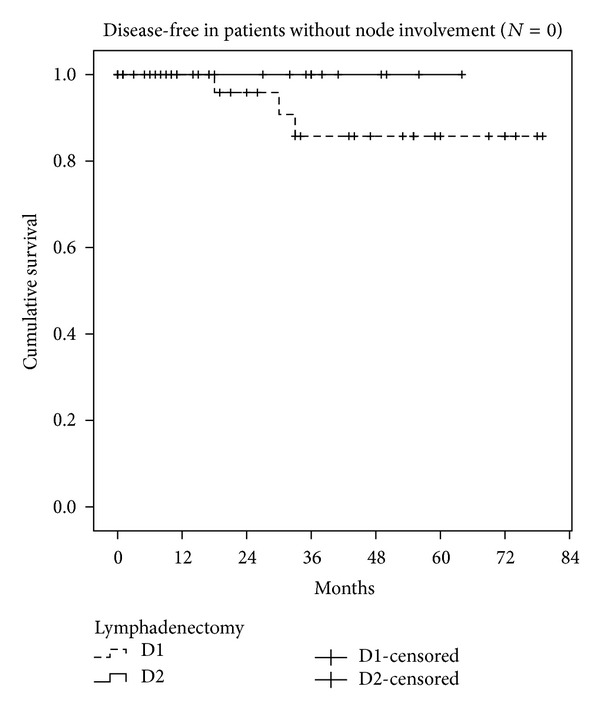
Survival in patients without lymph node involvement (*N* = 0), group D1 versus group D2.

**Figure 4:**
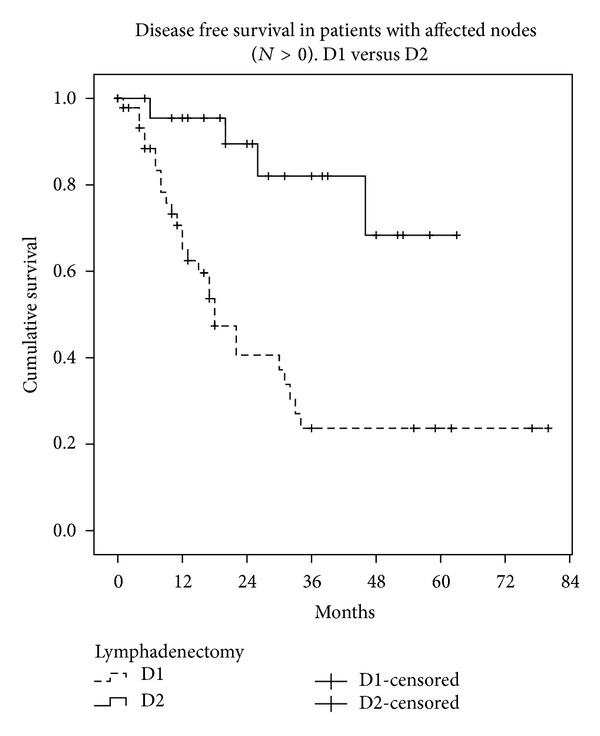
Survival in patients with lymph node involvement (*N* > 0), group D1 versus group D2.

We also studied the disease-free interval and overall survival in both groups (Figures 1 and 2). Median survival was 42.9 months in D1 (95% CI: 35–50 months) and 55 months in D2 (95% CI: 48–62 months, Kaplan-Meyer test, *P* < 0.001). The disease-free interval was 49 months in D1 (95% CI: 41–57 months) and 58 months in D2 (95% CI: 52–63 months, Kaplan-Meyer test, *P* < 0.001). One way to mitigate the limitations of the study design is to analyse only the survival of patients without lymph node involvement (*N*0) in both groups. Survival in these patients was similar (Kaplan-Meyer test, *P* = 0.152) (Figure 3), while patients with lymph node involvement (*N* > 0) in the D2 group had better survival than group D1 (Kaplan-Meyer test, *P* < 0.001) (Figure 4). 

The predictors of survival using Cox regression were: the node ratio (*B* = 1.3, exp (*B*) = 3.7, *P* = 0.045), the staging (*B* = 1.3, exp⁡(*B*) = 3.7, *P* < 0.001), and D2 lymphadenectomy (*B* = −1.7, exp⁡(*B*) = 0.18, *P* = 0.002). 

## 6. Discussion

There is convincing evidence from cohort studies that gastrectomy with D2 lymphadenectomy is associated with low morbidity and mortality when the resection of the spleen and pancreas is avoided. Therefore, there is no reason to conclude that the current form of D2 lymphadenectomy is more dangerous than D1 [3]. 

The recent meta-analysis published in Annals of Surgery suggested that the learning curve is a determining factor for morbidity and mortality [4]. In four of the six valid trials, specific training for D2 lymphadenectomy had neither been provided previously, nor was provided during the study itself. The results of the Italian study [3], in which the surgeons had already operated on 25 patients, are the most similar to those obtained in Japan. 

Prior to the start of the present study, our team of three surgeons had received training at the National Cancer Center in Tokyo, Japan. In 2006 we began to select patients for D2 lymphadenectomy, assessing BMI, age, comorbidity, and tumor stage. To date we have performed 48 gastrectomies with D2 lymphadenectomy, without any significant increase in morbidity. 

Comparing our results with those of other Western groups, our morbidity rate in D2 lymphadenectomy (33.9%) is lower than those reported in multicenter studies by Bonnenkamp [5] (43%) and Cuschieri [6] (46%) but higher than those observed by another Spanish group [9] (29%) and by the Italian group [3] (16.3%). These large discrepancies between groups are probably due to differences in patient selection and to differences in the point in the surgeons' learning curve at which the study was performed. 

Wu et al. [15] found decreased morbidity and mortality only after performing 200 radical gastrectomies. However, we believe that the learning curve may be much shorter and that morbidity and mortality rates are acceptable if the procedure is performed by experienced surgeons and with strict patient selection. 

The Japanese multicenter study JCOG9501 [16] found that overweight (BMI > 25) is a risk factor for prolonged operating time and complications, especially in the case of a D2 lymphadenectomy. The study by Kodera et al. [17] states that the risk factors for complications after gastrectomy with extended lymphadenectomy are BMI > 25, age > 65 years, and the performance of a pancreatectomy. Following these findings, our study included only patients aged under 70 and with BMI under 30, and pancreatectomies were avoided if there was no tumor infiltration. 

Although we are aware that our D1 and D2 groups are not comparable or randomized, the disease-free interval and overall survival in both groups are relevant findings. In the D2 group there have been no locoregional recurrences, and survival rates are encouraging. The results are very favorable to D2 lymphadenectomy but we emphasize that the study was not designed for the purpose of comparison and therefore does not have objective value.

One way to avoid the initial bias in the two groups is to carry out a post hoc analysis: that is, once we know whether the patient has node involvement or not, we assess their survival and then make comparisons between the two lymphadenectomy groups. We are well aware that this design does not allow a level of evidence above III, but this does not render it invalid in the absence of randomization. What we recorded is the survival of patients without lymph node involvement (*N*0) and with involvement (*N* > 1) in the two groups, finding similar survival rates in *N*0 and improved survival rates in *N* > 1 in the D2 lymphadenectomy group. The minimal difference (which did not reach statistical significance) observed in the D1 and D2 curves in patients with *N*0 can be attributed to understaging of patients undergoing D1 who were actually affected *N*1 but in whom the nodes responsible for recurrence and therefore for the shorter disease-free interval remained in the surgical bed. It seems logical that D2 lymphadenectomy does not achieve a great improvement in the survival of *N*0 patients, but the fact that it improves *N* > 1 patients could be due to the oncological role that extended lymphadenectomy may play, preventing the spread of gastric cancer through the lymphatic system. If we add that D2 lymphadenectomy may detect some skip metastases that pass unnoticed with the D1 technique, the decision to perform D2 seems justified, after a suitable learning period. 

We believe that strict, comprehensive monitoring of the adverse effects and morbidity associated with any technique is essential before making changes in clinical practice in a surgical department. A new surgical technique should only be introduced if it is clearly shown that it does not increase morbidity. To establish this, it is mandatory to monitor adverse effects continuously and to record the results in databases in order to track the evolution of complications over time [10, 18].

## 7. Conclusions

A demanding technique like D2 lymphadenectomy can be performed safely.

The improvement in surgical skill and the increased implication of the pathologist allow a gradual increase in the number of nodes isolated, even when a D1 lymphadenectomy is performed.

Extended lymphadenectomy may play an oncological role in gastric cancer.

## Figures and Tables

**Table 1:** Postoperative morbidity and mortality (compared with POSSUM) between January 2005 and December 2011.

	D1	D2
Observed morbidity	59.4%	47.7%
Expected morbidity (POSSUM)	49.4%	33.7%
Ratio morbidity	**1.202**	**1.415**

Observed mortality	6.7%	6.8%
Expected mortality (O-POSSUM)	13.9%	7.2%
Ratio mortality	**0.482**	**0.944**

**Table 2:** Description of postoperative complications (according to Clavien). 89 patients with lymphadenectomy D1 and 44 with D2. Pancreatic fistula described by the ISGPF classification [14].

CLAVIEN	D1	D2
Grade I: no action		
Evisceration	5	1
Postoperative bleeding		1
Pancreatic fistula grade A		1
Grade II requires medication		
Intra-abdominal abscess	9	2
Central venous catheter-related infection	4	
Anastomotic leakage	4	
Urinary tract infection	3	
Pneumonia	3	1
Surgical wound infection	2	2
Pancreatic fistula grade B		1
Pancreatitis		1
Grade IIIa requires intervention without general anesthesia		
Surgical wound infection	6	1
Intra-abdominal abscess	6	
Anastomotic leakage	2	
Grade IIIb requires intervention with general anesthesia		
Anastomotic leakage	4	1
Evisceration	2	
Intra-abdominal abscess	2	
Wound bleeding	1	1
Postoperative bleeding		2
Pancreatic fistula grade C		1
Intestinal occlusion		1
Grade IVa: organ failure-ICU		
Acute myocardial infarction	1	3
Pneumonia	1	
Grade IVb: multiorgan failure-ICU		
Acute myocardial infarction	1	
Iatrogenic colon perforation		1
Pneumonia		1
Grade V: death		
Cardiac arrhythmia	1	
Multiorgan failure	3	1
Heart failure	1	
Kidney failure	1	
Liver failure		1
Postsurgical pancreatitis		1
